# Stress-Driven Tolerance and Persistence of *Listeria monocytogenes* Across the Farm-to-Fork Continuum

**DOI:** 10.3390/biology15040310

**Published:** 2026-02-10

**Authors:** Ayman Elbehiry, Eman Marzouk, Adil Abalkhail

**Affiliations:** Department of Public Health, College of Applied Medical Sciences, Qassim University, P.O. Box 6666, Buraydah 51452, Saudi Arabiae.marzouk@qu.edu.sa (E.M.)

**Keywords:** sublethal stress, *Listeria monocytogenes*, dormancy, stress response, microbial persistence, detection limitations, food safety, public health

## Abstract

Food systems expose bacteria to repeated nonlethal stresses during primary production, processing, storage, and sanitation. Depending on the type, intensity, and sequence of exposure, these stresses may weaken cells, act synergistically to promote inactivation, or fail to eliminate contamination. Instead, they can alter bacterial physiology in ways that affect survival, recovery, detection, and responses to control measures. This review examines how stress history contributes to persistent food safety challenges. *Listeria monocytogenes* is used as a central biological model, with relevant comparisons to other foodborne pathogens. Evidence from food-processing and environmental studies shows that repeated sublethal stress can shift bacterial populations toward stress-hardened states. Here, “stress-hardened” refers to reversible physiological changes and the survival of more tolerant cells, not permanent genetic adaptation. These states include sublethal injury, delayed growth, viable but nonculturable cells, biofilm formation, and increased tolerance to later stresses. These outcomes contribute to, but do not fully explain, the persistence of *L. monocytogenes* in food environments; intrinsic traits such as psychrotrophic growth and interactions with endogenous microflora also play important roles. These factors help explain repeated recovery of *L. monocytogenes* after sanitation and the underestimation of viable cells by routine culture-based methods, which do not reliably indicate whether pre-stressed cells retain the potential to cause foodborne illness. Many monitoring and validation approaches rely on unstressed laboratory cultures and fixed enrichment protocols. These conditions do not reflect the physiological states encountered in real food systems. As a result, negative test results may reflect limited recovery rather than true absence, and control performance may be overestimated when stress-conditioned populations are not considered. Across the farm-to-fork continuum, stress responses, persistence mechanisms, and detection limitations are closely linked, indicating that stress history should be considered a core element of hazard characterization, monitoring, and control validation. Incorporating stress biology into food safety assessment can improve the realism of verification strategies when combined with risk characterization that considers infectious dose and host susceptibility, and support control strategies under real-world processing and environmental conditions.

## Simple Summary

As food moves from farms to consumers, bacteria are exposed to many everyday stresses. These include refrigeration, drying, cleaning chemicals, and food processing steps. These stresses do not always kill bacteria. Instead, they can change how bacteria behave. Some cells recover slowly, hide in biofilms, or enter dormant states that routine tests cannot easily detect. This review explains how repeated stress affects bacterial survival, using Listeria monocytogenes as a key example. We show how stress history helps explain why Listeria can persist in food-processing environments, reappear after cleaning, and sometimes escape detection. Understanding how bacteria respond to stress in real food systems can improve monitoring, sanitation, and food safety decisions.

## 1. Introduction

Food safety remains a major public health concern because microbial contamination continues to occur across a wide range of foods and production systems [1]. The World Health Organization estimates that unsafe food causes approximately 600 million illnesses and 420,000 deaths each year worldwide [2]. Beyond health impacts, unsafe food also leads to substantial economic losses, particularly in low- and middle-income settings, through increased healthcare costs and reduced productivity [2,3]. Among the pathogens responsible for this burden, those able to survive food processing and persist in production environments present particular challenges for control in food production and processing systems.

*Listeria monocytogenes* is a Gram-positive facultatively anaerobic foodborne bacterium responsible for listeriosis in humans and animals [4,5]. Although listeriosis is less common than many other foodborne diseases, it is associated with high hospitalization and mortality rates. Reported case fatality rates range from approximately 20 to 30% in clinical cases worldwide [6]. *L. monocytogenes* is repeatedly detected in dairy products, meat, fresh produce, and ready-to-eat (RTE) foods. These detections have led to recurrent outbreaks and product recalls, creating ongoing public health concerns [7,8,9].

Once introduced into food production and processing environments, *L. monocytogenes* can survive under conditions that inhibit the growth of many competing microorganisms [10]. These conditions include refrigeration temperatures, acidic environments, and elevated salt concentrations [6,10]. The bacterium can also attach to surfaces and form biofilms on food-contact materials. Biofilm formation protects cells from environmental stress and routine sanitation, making eradication difficult [11]. In food processing environments, *L. monocytogenes* commonly occurs within mixed-species biofilms, where resident microflora can enhance survival by providing physical protection and modifying local conditions [12,13]. Persistence of *L. monocytogenes* in processing environments has been documented for months to years and is often linked to repeated food contamination and regulatory challenges [14].

During food production, processing, and sanitization, *L. monocytogenes* cells are repeatedly exposed to sublethal physical and chemical stresses. These stresses include acid exposure, disinfectants, low temperature, and osmotic pressure [15,16]. Depending on their intensity and combination, such stresses may be lethal for part of the population. Rather than causing immediate inactivation, such conditions can induce sublethal injury or entry into the viable but nonculturable (VBNC) state. In this state, cells remain viable but do not grow under standard culture conditions [16]. Resuscitation has been observed when stress is removed and cells are provided with nutrients, moderate temperatures, and nonselective recovery conditions [17]. These stress-induced states reduce recovery in routine culture-based detection methods and allow survival under conditions that would otherwise limit growth or cause cell death [15].

Transitions between stressed, sublethally injured, and VBNC states allow *L. monocytogenes* to tolerate repeated environmental challenges encountered in food systems [18,19]. These responses involve documented changes in membrane integrity, metabolic activity, and gene expression. Overall, these changes increase tolerance to subsequent stresses applied during food processing and sanitation [20,21,22]. As a result, stress-exposed populations can persist in food environments and complicate microbiological assessment by escaping routine culture-based detection [23,24,25].

In this review, we examine how repeated exposure to sublethal stress during food production and processing influences the survival, persistence, detection, and control of *L. monocytogenes*. The review focuses on documented physiological responses that are directly relevant to food safety practice and regulatory monitoring. By integrating mechanistic studies with observations from food-processing environments, this review explains why contamination by *L. monocytogenes* can persist despite established intervention strategies.

This narrative review is based on peer-reviewed studies identified through searches of Web of Science, PubMed, and Scopus. The literature was selected to cover food-relevant stress exposure, persistence, detection, and control of foodborne bacteria, with emphasis on studies published over the past two decades. *L. monocytogenes* was chosen as the main model organism because it is well studied, frequently associated with food processing environments, and supported by extensive field and laboratory evidence that links stress exposure to persistence and control failure. Evidence from other foodborne pathogens is included where it supports or contrasts with patterns observed for *L. monocytogenes*.

**Figure 1:**
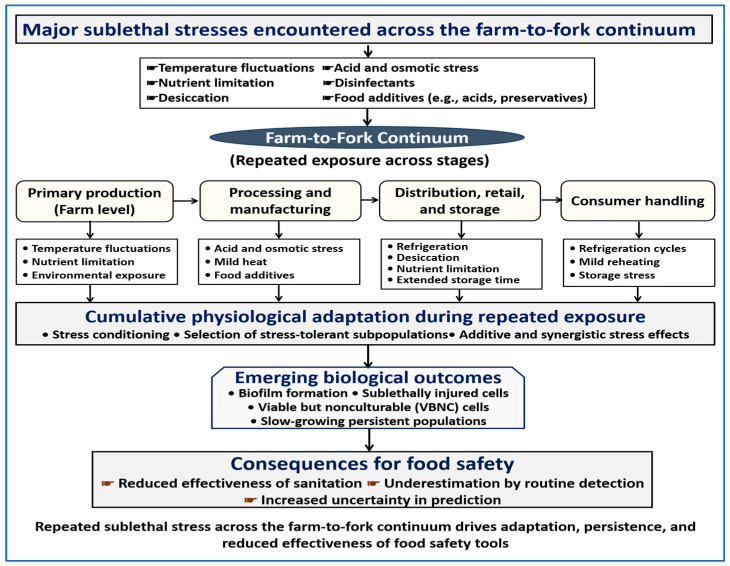
System-level framework illustrating repeated sublethal stress exposure across the farm-to-fork continuum. *L. monocytogenes* encounters multiple stresses during primary production, processing and manufacturing, distribution, retail and storage, and consumer handling, including temperature fluctuations, desiccation, disinfectants, food additives, nutrient limitation, and extended storage time. These stresses act cumulatively and may combine additively or synergistically rather than occurring as isolated events. Repeated exposure promotes physiological adaptation and selection of more tolerant subpopulations, leading to persistence-enabling outcomes such as biofilm formation, sublethal injury, VBNC cells, and slow-growing persistent populations. These outcomes reduce sanitation effectiveness, limit recovery by routine detection methods, and increase uncertainty in food safety assessment.

Figure 1 summarizes the framework used in this review. It shows how *L. monocytogenes* is exposed to successive stages of the food chain as a series of repeated sublethal stresses rather than as single, isolated control steps. As cells move from primary production through processing, distribution, and consumer handling, repeated exposures drive physiological adaptation. Over time, this process favors bacterial subpopulations with increased tolerance to stress, including biofilm-forming cells, sublethally injured cells, VBNC cells, and slow-growing persistent populations. These features help explain recurrent contamination and the reduced effectiveness of sanitation, routine detection, and predictive tools in real food systems.

## 2. Rethinking Food Safety Through Bacterial Biology

Food safety is commonly framed as a sequence of control steps, including cleaning, disinfection, processing, and monitoring, that are intended to reduce microbial hazards [26,27]. However, in real food systems, pathogens such as *L. monocytogenes* are often exposed to sublethal stresses during production and processing. Many food preservation and sanitation practices are designed to limit microbial growth or reduce contamination rather than to cause immediate inactivation [23,28]. In response, the bacterium activates stress response pathways and changes gene expression [29]. Sublethal acid, oxidative, or disinfectant stresses can lead to physiological states such as sublethal injury or the VBNC state. These states support survival during later challenges in food environments [30,31]. These states have been demonstrated primarily under controlled laboratory conditions and explain how cells can persist under food-relevant stress, rather than serving as direct evidence of persistence in commercial settings.

Stress adaptation in *L. monocytogenes* provides a clear example. During production, processing, storage, distribution, and preparation, the bacterium encounters changes in temperature, pH, osmotic pressure, drying, and exposure to sanitizers [23]. Begley and Hill showed that bacteria sense these changes and adjust the expression of stress response genes. They also demonstrated that these responses can increase tolerance to later stresses [29]. In laboratory studies, stress adaptation has also been shown to influence traits linked to host interaction after ingestion, depending on the experimental model used [32].

A practical implication of stress adaptation is reduced effectiveness of later control steps, as prior stress exposure can increase tolerance to different stresses [33]. Leyer and Johnson showed that acid adaptation in *Salmonella typhimurium* increased resistance to several environmental stresses. Their work illustrates a key principle for food systems [34]. Validation guidelines such as those from NACMCF use controlled challenge studies under defined conditions. However, these studies do not reflect the varied and repeated stress exposures that bacteria experience in real food production and processing environments [35].

Sublethal injury makes *L. monocytogenes* harder to control because processing and sanitation can harm cells without killing them, allowing them to recover when conditions improve [30]. Wesche and colleagues reviewed how stress and injury occur and how repair takes place. They also showed that prior stress exposure can lead to cross-protection. Some stress conditions may also influence traits related to virulence, which becomes important when surviving cells reach the consumer [30].

Persistence of *L. monocytogenes* in food processing facilities reflects its ability to survive and re-establish in the same environment over time. As a result, contamination may reoccur even when hygiene practices remain unchanged [36]. One reason is surface attachment and biofilm formation. Møretrø and Langsrud showed that *L. monocytogenes* forms biofilms that protect cells from environmental stress [37]. They also reported recovery of cells from surfaces after cleaning and disinfection, which helps explain why eradication becomes difficult once persistence is established [11].

Monitoring results for *L. monocytogenes* can be misleading when stress exposure alters bacterial physiology [17]. Highmore and colleagues showed, using a laboratory model, that chlorine exposure induced a VBNC state in *L. monocytogenes* and *Salmonella enterica* and that these cells retained infectivity in *Caenorhabditis elegans* despite loss of culturability [31]. These findings demonstrate a disconnect between culturability and biological activity in experimental systems but do not represent direct evidence of transmission or disease in food-processing environments.

Predictive microbiology is strongly influenced by how bacteria respond to environmental conditions and physiological states, which affect model predictions of growth and inactivation in foods [38]. Predictive models are useful for estimating microbial growth and inactivation under defined conditions [39,40]. Buchanan described how such models are used to represent microbial behavior in foods [41]. In real food systems, *L. monocytogenes* encounters changing stresses (temperature, acid, salt, oxidants, drying, sanitizers) and can occupy multiple physiological states (sublethally injured, biofilm-associated, persisters, VBNC) [15,29]. Because models are commonly parameterized using unstressed or average populations, pre-stressed cells may be predicted as slow-growing or non-growing [30,39,41]. If stressed subpopulations recover under favorable conditions, growth potential may be underestimated, which reduces how well average model predictions match observed outcomes [42].

Food safety should therefore be understood as a biological process, not only as a procedural set of control steps. Repeated sublethal stresses, such as exposure to sanitizers, acids, and osmotic conditions, can alter how *L. monocytogenes* behaves over time [33]. Hygiene, processing, and monitoring remain essential, but their effectiveness can vary as stress-adapted populations develop along the farm-to-fork continuum (Figure 1).

## 3. The Food Chain as a Continuous Selective Environment

The food chain exposes bacteria to a series of changing conditions rather than to a single lethal event. Many of these conditions slow growth or damage cells without causing death. As a result, bacteria face repeated survival challenges across time and place [43]. From a biological standpoint, repeated exposure to mild stress favors survival over immediate elimination. Cells that tolerate these conditions are therefore more likely to persist and reach later stages of the food chain [44].

This view is consistent with the concept of hurdle technology. Food safety and stability are achieved through combinations of preservation factors rather than reliance on a single intervention [45]. Leistner emphasized that these hurdles are often adjusted to maintain food quality while limiting microbial growth [43]. Hurdle technology therefore operates at the level of microbial populations. Food safety is achieved through the additive and sometimes synergistic effects of multiple preservation factors, rather than through complete inactivation of individual cells [43,45]. Consequently, microorganisms are frequently exposed to inhibitory but sublethal conditions. This is especially true when temperature, pH, and water activity change during processing and storage [43]. Under such combined stress conditions, population-level selection and survival dynamics become more important than elimination by any single hurdle [43,46].

Food processing facilities also function as selective environments. Microorganisms on food-contact surfaces and in hard-to-clean areas are repeatedly exposed to nutrients, refrigeration, drying, and cleaning agents [46]. Møretrø and Langsrud described biofilm formation as a key survival strategy in food processing environments. They showed that biofilm-associated cells are protected against cleaning and disinfection and are difficult to eliminate once established [37]. In this setting, persistence reflects repeated environmental exposure rather than a single failure in hygiene.

Evidence from processing environments supports this interpretation. Carpentier and Cerf reviewed the persistence of *L. monocytogenes* in food industry equipment and premises. They reported that persistence is commonly linked to traits such as surface attachment, biofilm formation, resistance to drying, tolerance to acid and heat, and survival at sublethal disinfectant concentrations. These findings span a wide range of food processing environments, including dairy plants, meat and poultry processing facilities, RTE food operations, and fresh-produce packing and processing lines [36,46]. They also demonstrated that contamination is often associated with cold and wet areas, including floors, drains, refrigerated rooms, and equipment. These niches provide conditions that support long-term survival and repeated contamination [36].

Cleaning and disinfection can further contribute to selection when exposure is incomplete or when protective microenvironments are present [11]. Fagerlund and colleagues examined biofilms from meat-processing surfaces and showed that bacteria can survive foam cleaning and disinfection at concentrations commonly used in industrial practice. They reproduced this survival under laboratory conditions, indicating that persistence reflects biological tolerance rather than accidental failure [47]. Traits linked to survival included growth at low temperature, tolerance to cleaning agents, and the ability to form biofilms. These traits are consistent with selection during repeated hygiene cycles [47].

Selection is also shaped by stress history. Earlier exposure can change how bacteria respond to later hurdles. In *L. monocytogenes*, acid adaptation and stress regulation are linked to improved survival under different stress conditions [48]. The alternative sigma factor σ^B^ plays a central role in survival during both lethal and sublethal stress [49]. More recent studies show that gradual exposure to sublethal disinfectant concentrations can modify later stress responses. This reflects the repeated oxidative stress encountered during routine sanitation [20].

Highmore and colleagues demonstrated, in a laboratory nematode model, that chlorine exposure induced VBNC cells and reduced host lifespan after ingestion [31]. However, available evidence does not establish that stressed or VBNC cells have the same infectious dose as unstressed cells, and the dose–response relationship for such cells remains uncertain and context dependent [17,19,50]. These findings show that culturability and biological activity do not always align in food systems. Importantly, culture-independent molecular methods can detect VBNC cells and therefore provide additional insight into viable populations that may be missed by routine culture-based diagnostics [51,52].This result highlights potential risk mechanisms under experimental conditions and supports caution when interpreting negative culture results, rather than serving as direct field evidence.

Overall, the farm-to-fork continuum functions as a continuous selective environment. Combined hurdles applied to foods, repeated hygiene cycles in processing facilities, and stress-driven physiological changes can enrich bacterial subpopulations that tolerate later interventions. These populations are more likely to persist in niches that are difficult to control [36,44,48].

## 4. How Bacteria Respond to Stress in Food Systems

### 4.1. Physiological Responses to Repeated Stress

Foodborne bacteria move through environments that change repeatedly during production, processing, storage, and handling [53]. Common conditions include temperature shifts, changes in pH, osmotic stress, limited nutrients, and exposure to cleaning chemicals. These exposures do not always kill bacterial cells. Instead, they often trigger physiological responses that allow survival until conditions improve [24,25].

Stress responses are not limited to a single species. A detailed review of non-typhoidal *Salmonella* shows that bacteria encounter many stresses in food and processing environments, including acid, salt, oxidative stress, nutrient limitation, cold, heat, detergents, and disinfectants. These conditions shape bacterial physiology and survival. The review also shows that stress responses can influence traits linked to virulence, which becomes important when surviving cells reach the consumer [24].

In food systems, stresses are usually combined and repeated rather than isolated. Cells are therefore challenged in cycles [54]. Repeated exposure increases the chance that part of the population survives in an altered physiological state rather than returning to normal growth. This provides a biological explanation for why tolerance and persistence can emerge even when individual control steps appear effective [24,25].

### 4.2. Global Stress Regulation and Adaptive Flexibility

A key feature of bacterial survival under food-related stress is the use of global regulatory systems that coordinate protective functions [55]. In *L. monocytogenes*, the alternative sigma factor σ^B^ controls a broad general stress response [50]. Its activation depends on a stress-sensing complex that integrates environmental signals. A focused mini-review describes how σ^B^ contributes to stress tolerance and how its activity is regulated in response to environmental stress [56].

Activation of the general stress response improves survival but also carries a cost. Stress responses require cellular resources, which limits growth and metabolic efficiency. This trade-off is especially relevant in foods, where conditions often restrict growth without eliminating cells [56].

Recent synthesis work places this regulatory flexibility in food system contexts. A comprehensive review of *L. monocytogenes* adaptation describes how stress responses support survival during food processing and storage. It also explains how σ^B^ and other regulatory elements are controlled under food-relevant conditions. Proteomic and transcriptomic evidence summarized in that review shows activation of stress-responsive pathways under conditions that resemble food environments [25].

Global stress regulation is also well described in Gram-negative pathogens. A review of *Salmonella* shows that resistance responses developed under food-associated stresses shape survival and growth across multiple conditions. In this organism, the sigma factor RpoS functions as a master regulator of the general stationary-phase stress response and supports survival during prolonged or sublethal stress [24,57]. These examples show that global regulators promote flexibility when stress is persistent rather than lethal.

### 4.3. Cross-Protection and Stress-Induced Tolerance

In food systems, stresses usually occur in sequence. One consequence is cross-protection, in which exposure to one sublethal stress increases tolerance to a different stress encountered later [48]. An experimental study showed that acid adaptation induced cross-protection against several environmental stresses in *S. typhimurium*. This work demonstrated that exposure history can alter later stress resistance [34].

Cross-protection is important because food control measures are often tested using bacteria without prior stress exposure. This does not reflect real food systems. In practice, bacteria encounter multiple stresses during production, storage, and transport before processing begins. These early exposures can change physiology and increase tolerance to later interventions. Several studies show that mild or sublethal stress can increase resistance to treatments that would otherwise be lethal, a phenomenon known as cross-protection [58]. As a result, interventions that perform well under laboratory conditions may show variable effectiveness in food environments [59].

Recent work shows that tolerance is not always fixed before processing. A 2025 study by Guillén et al. examined bacterial responses during dynamic heat treatments that resemble real processing conditions. They found that cells could adapt physiologically while heating was still in progress. This gradual adaptation increased heat tolerance compared with static or abruptly applied treatments [60]. Related work showed that nonlinear survival curves can arise from dynamic stress adaptation during treatment, not only from pre-existing differences among cells [61]. These findings highlight the importance of exposure sequence and timing when designing or validating control steps.

### 4.4. Phenotypic Diversity Within Stressed Populations

Even within a single strain, bacterial populations do not respond uniformly to stress. Cells differ in physiological state and readiness to respond. As a result, a small fraction may survive treatments that inactivate most of the population. A 2022 review by Martin et al. describes genetic, physiological, and cellular heterogeneity of foodborne pathogens and explains how food structure and microenvironments create spatial and temporal phenotypic diversity [62].

This diversity contributes to variability in inactivation outcomes, even when processing conditions appear similar. A review of microbial inactivation variability shows that describing survival as a distribution of single-cell outcomes better reflects reality than relying on average behavior alone [63].

Phenotypic diversity also complicates detection and interpretation after stress exposure. Chlorine has been shown to induce a VBNC state in *L. monocytogenes* and *S. enterica*, with retained biological activity demonstrated in the *Caenorhabditis elegans* infection model [31]. These findings indicate that loss of culturability does not necessarily reflect loss of biological function, but they should be interpreted strictly within the limits of the experimental model and not as direct evidence of human infectivity. A later review focused on the VBNC state of *L. monocytogenes* describes its formation in environmental, agricultural, and food industry settings and highlights the role of antimicrobial treatments in induction [18].

Recent experimental work continues to link oxidizing sanitizers with VBNC induction and persistence traits in *Listeria*. A 2025 study reported that free chlorine induced the VBNC state and persistence in *L. monocytogenes* and described the emergence of high-fitness phenotypic variants under this stress [64]. Variability is not only biological. When survival probabilities are low, chance also influences outcomes. Garre and colleagues showed that stochastic effects shape survivor curves, particularly when persistence depends on rare surviving cells [65].

Global stress regulation, cross-protection, and phenotypic diversity together explain why bacterial survival varies in practice, even when hygiene and processing are correctly applied. These mechanisms account for the persistence of stress-hardened fractions across multiple stages of the food chain and explain why average inactivation values do not reliably predict real-world outcomes [25,34,56,62].

## 5. Persistence Outcomes of Stress-Hardened Bacteria in Food Systems

Stress responses do not end when stressful conditions are removed. In food systems, the key issue is the fate of cells that survive sublethal exposure [29]. During production, processing, and storage, bacteria are repeatedly exposed to sublethal stress. Over time, this exposure can shift populations toward physiological states that are harder to eliminate, harder to detect, and more likely to reappear [29]. Persistence outcomes of greatest practical relevance include surface-associated growth in biofilms, survival as injured or slow-growing cells, and entry into VBNC states [16,66,67]. Entry into the VBNC state can occur through both stress-induced physiological transitions in individual cells and selective enrichment of stress-tolerant subpopulations, depending on the type, intensity, and history of exposure [17,68,69].These outcomes are well recognized for *L. monocytogenes* and provide a useful model for understanding stress-hardened persistence in food environments.

### 5.1. Biofilm Formation as a Persistence Strategy

Biofilm formation is a common outcome of stress-hardened survival. In food-processing environments, biofilms develop on food-contact materials and in niches where moisture, nutrients, and incomplete removal during sanitation permit attachment and growth [70]. A recent review described biofilms in food-processing settings as reservoirs that may contain major foodborne pathogens and showed their formation on materials such as stainless steel, plastics, and rubber [66]. Within biofilms, bacteria live close together and are held in place by a shared matrix that surrounds the cells [71]. This structure limits how easily disinfectants and other stresses reach individual cells, which increases survival compared with free-living bacteria [72]. Biofilms also create uneven access to nutrients and oxygen, leading to mixed populations that include slow-growing and more stress-tolerant cells [73].

Once established, biofilms change how bacteria respond to sanitizers. In *L. monocytogenes*, mature biofilms are less susceptible to commonly used disinfectants [74,75]. This is directly relevant to repeated contamination events in food supply chains. Sanitation programs are often evaluated using free-living cells, whereas contamination in practice frequently involves surface-associated communities [76].

Biofilm-forming ability also varies among industrial isolates. A study of *L. monocytogenes* from meat-processing environments reported clear differences in biofilm formation between strains. These findings demonstrate that persistence risk depends partly on the characteristics of the strains present within a facility [77].

Effective control of *L. monocytogenes* biofilms requires multiple disinfection approaches and attention to the conditions that promote attachment and regrowth [11]. These approaches include the use of chemical disinfectants with different modes of action, combined with thorough physical cleaning to remove attached cells and biofilm material [36]. Effective control also depends on sanitation frequency, sufficient contact time, and management of moisture and nutrient residues that support biofilm regrowth [78]. This supports a central point of this review: biofilms are not separate from stress adaptation but represent one outcome selected by repeated exposure across the farm-to-fork continuum [66,79].

Biofilm-linked persistence is not limited to *Listeria*. Studies of *S. enterica* from food production chains show that biofilm formation can coincide with increased sanitizer tolerance, including among strains associated with specific commodities [80]. Similar findings have been reported for *Escherichia coli* (*E. coli*) O157:H7, where strains linked to high-event contamination periods in beef processing formed stronger biofilms and showed greater resistance to sanitation [81]. Food-processing-focused studies further demonstrate that biofilm-associated cells of *L. monocytogenes*, *E. coli* O157:H7, and *Salmonella* serovars can survive treatments commonly used in industrial practice [82,83].

### 5.2. Injured, Slow-Growing, and Dormant Cells

Sublethal treatments often cause cellular injury rather than immediate death. Injured cells may show membrane damage, impaired metabolism, or oxidative stress while remaining viable and capable of recovery under favorable conditions [84]. A comprehensive review published in 2023 summarized how sublethally injured microorganisms arise during food processing, how they can be detected, and how resuscitation occurs [67]. This review builds on earlier studies to show how repeated, low-level stresses across the food chain allow bacteria to persist, make detection harder, and weaken control efforts in real food systems.

Cell injury is important because it directly affects monitoring outcomes. Culture-based methods may underestimate contamination when sublethally injured cells fail to grow under selective enrichment conditions [30,85]. Such cells are often sensitive to selective agents, elevated salt, low pH, or oxidative stress and may only recover on non-selective media, after extended incubation, or under modified atmospheric conditions [85]. These effects have been well documented in food microbiology and explain how recovery protocols strongly influence whether injured cells are detected during routine monitoring [86].

Recent work has examined these issues in realistic food contexts. A 2025 study evaluated thermal injury of *L. monocytogenes* on RTE meat following mild post-packaging heat treatment and compared physiological status at both single-cell and population levels. These results are relevant because mild treatments can be sublethal for cells in protected niches, producing survivors that differ from untreated populations [87].

Beyond injury, food systems can also select for slow-growing or dormant phenotypes. A 2024 review of fresh-produce supply chains discussed dormancy and identified conditions that promote dormant cell formation along the chain. This work frames persistence as a physiological strategy suited to environments with fluctuating stress and limited nutrients [88]. A 2025 review further described small-colony variants as slow-growing phenotypes with altered metabolism that can persist in foods and processing environments and increase contamination risk [89].

Evidence for injury and recovery is not limited to *Listeria*. In processing-relevant settings, biofilm-associated *E. coli* O157:H7 and *Salmonella* have been used to evaluate post-sanitization survival and recovery under conditions designed to reflect industrial cleaning practices [82].

Bacteria can enter dormant states that allow survival during prolonged stress without active growth [90,91]. Dormancy includes persister cells, small-colony variants, and the VBNC state, all of which reduce culturability while often maintaining metabolic activity [17]. These states can be triggered by stressors common along the food chain, including cold storage, drying, acidic conditions, and exposure to disinfectants. They may reverse when conditions become favorable, allowing cells to resuscitate and contribute to later contamination [17,92]. Mixed-species biofilms and resident microflora further influence dormancy by providing physical protection and by modifying local conditions that favor non-growing states [37,78]. Overall, dormancy and microbial community context complicate detection, verification, and control, particularly when monitoring relies on growth-based methods that fail to capture viable but non-growing cells [17,91].

### 5.3. Viable but Nonculturable States

The VBNC state represents one of the most challenging persistence outcomes for food safety because it directly limits routine verification [93]. In this state, bacteria fail to form colonies on standard media while remaining viable and potentially active. VBNC is one of several dormancy-related survival strategies, alongside persister cells and other metabolically slowed or dormant subpopulations that remain viable without active growth [68,90,94].

At the genetic level, entry into the VBNC state is not driven by a single gene or switch. Instead, it reflects broad changes in stress-related gene regulation that help cells conserve energy and cope with damage [17,68]. These changes involve global stress regulators and pathways linked to oxidative stress defense, metabolism, and survival, and they vary depending on the organism and the type of stress encountered [69,94]. VBNC should therefore be viewed as a stress-driven physiological state rather than a fixed, genetically programmed form of dormancy [17,68].

A 2018 study showed that chlorine stress induced VBNC *L. monocytogenes* and *S. enterica* and reported that these cells remained infectious in a model system [31]. VBNC formation is induced by multiple stressors. A 2025 review focused on VBNC *L. monocytogenes* summarized known inducing conditions and discussed implications for food safety practice, including prevention strategies and remaining knowledge gaps [16].

VBNC induction and recovery have also been examined under combined environmental pressures relevant to food chains. A 2024 experimental study induced the VBNC state in *L. monocytogenes* using combinations of temperature stress and nutrient limitation and evaluated recovery behavior. These findings are relevant because such conditions are common during storage, distribution, and processing [95].

VBNC formation is also reported in other food-relevant pathogens. Studies on *Campylobacter jejuni* show that stresses common in agrifood systems can reduce culturability while maintaining viability, raising concerns about detection failure [96]. Similar evidence exists for *Vibrio parahaemolyticus*, which has been shown to enter the VBNC state under cold-starvation conditions relevant to seafood storage [97]. Recovery of culturability after temperature upshift further highlights the role of environmental transitions during distribution and preparation [98].

These persistence outcomes explain why stress-hardened bacteria remain difficult to control even in well-managed food systems. Biofilms protect cells and promote recurring contamination. Injured and slow-growing populations complicate both inactivation and detection. VBNC formation allows biologically active cells to escape routine culture-based monitoring. Overall, outcomes most extensively characterized in *L. monocytogenes* illustrate how stress biology translates into persistence across the food chain [31,66,67].

### 5.4. What L. monocytogenes Teaches Us About Persistence in RTE and Processing Environments

*L. monocytogenes* provides one of the clearest and best-documented examples of how stress-hardened persistence develops in real food-processing environments [36]. Unlike many foodborne pathogens, it can survive repeated exposure to cold, acid, salt, and disinfectants commonly encountered in RTE production facilities [48,99]. This combination of stress tolerance and environmental survival helps explain why *L. monocytogenes* is repeatedly recovered from the same facilities over long periods, even when hygiene practices remain unchanged [36,100].

Studies from dairy, meat, and RTE processing plants show that persistent *L. monocytogenes* strains are often associated with specific niches such as drains, floors, conveyor belts, and hard-to-clean equipment, including slicers and cutters [36,101,102,103]. In these locations, biofilm formation, sublethal injury, and delayed recovery work together to protect cells from sanitation and reduce detection by routine monitoring methods [74,104]. These traits allow small subpopulations to survive cleaning, recover after sanitation, and contaminate products intermittently rather than continuously.

Because these persistence mechanisms have been studied in detail for *L. monocytogenes*, they provide practical lessons for food safety management [36]. They show that persistence is not driven by a single failure but by repeated sublethal exposure across the farm-to-fork chain [29]. The *Listeria* model therefore helps explain why control, detection, and prediction can fail in RTE food processing environments when stress history is not considered [46]. Table 1 summarizes key persistence mechanisms of *L. monocytogenes* that are directly relevant to food processing and RTE food processing environments.

## 6. Detection and Monitoring Limitations in the Presence of Stress-Hardened Bacteria

Stress-hardened bacteria pose a practical challenge for routine food safety verification. Many monitoring programs assume that target cells are culturable, recover quickly, and respond predictably to selective enrichment [19,67]. These assumptions hold for actively growing cells but often fail when cells are injured, slow to recover, or protected on surfaces. As a result, monitoring tends to detect cells that grow quickly under laboratory conditions, while cells that persist in foods or processing environments may be missed [67,108,109]. Culture-independent molecular diagnostic methods can detect non-growing or non-culturable bacterial cells and therefore complement traditional culture-based monitoring approaches [51]. However, these methods alone cannot distinguish between viable, inactive, or potentially recoverable cells without additional viability or physiological assessment [52].

**Figure 2:**
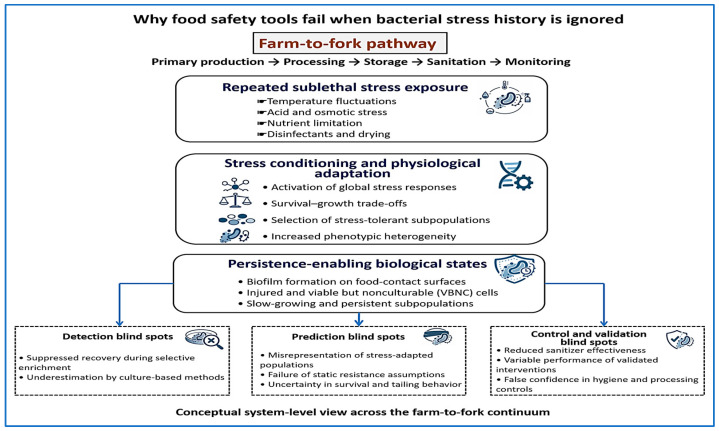
Why food safety tools fail when bacterial stress history is ignored. Repeated sublethal stresses encountered across the farm-to-fork continuum condition bacterial populations and drive physiological adaptation. These responses include activation of global stress systems, survival–growth trade-offs, and increased phenotypic heterogeneity. Over time, bacteria enter persistence-enabling states such as biofilm formation, sublethal injury, VBNC states, and slow-growing subpopulations. These states create blind spots in routine detection, prediction, and control validation, leading to underestimation of viable bacteria and variable performance of sanitation and processing interventions.

Figure 2 provides a conceptual overview of how repeated sublethal stress leads to adaptation, persistence, and blind spots in food safety tools. Spatial progression along the farm-to-fork continuum is illustrated in Figure 1. To support this framework, Table 2 summarizes common food-chain stressors, the persistence states they promote, and the corresponding detection blind spots observed during routine monitoring.

### 6.1. Culture-Based Detection and Its Blind Spots

Culture-based methods remain central to regulatory and industrial testing because they confirm growth and allow recovery of isolates [112]. Successful detection depends on whether stressed cells can repair damage and resume division under the enrichment and plating conditions used [113]. Reviews of sublethal injury show that injured cells may remain viable yet fail to grow on selective media, with recovery strongly influenced by the conditions provided [84,114].

Selective enrichment can therefore influence detection outcomes. For *Listeria* detection, ISO 11290-1 specifies staged enrichment with selective agents and fixed incubation times. Although standardized, method performance depends on how rapidly stressed cells recover during early enrichment [110]. Recovery is not uniform across strains or stress types. Studies show that lag time in half-Fraser broth varies among *L. monocytogenes* strains, and delayed recovery can prevent cells from reaching detectable levels within the enrichment schedule, leading to false-negative results [109]. Single-cell analyses further demonstrate that stress increases variability in lag time, allowing only a fraction of survivors to grow within the detection window [115].

In practice, the primary enrichment step is the most vulnerable stage for under-recovery of injured *L. monocytogenes* cells. Selective components added early can suppress repair before normal growth resumes [30,84]. The ISO 11290-1 protocol partly addresses this limitation by using half-Fraser broth, which applies reduced selectivity to support repair before full selective pressure [110]. Because recovery time differs among strains and stress histories, extended incubation or delayed transition to secondary enrichment may be needed in some cases [44,116]. When prior stress exposure is likely, laboratories may also use short non-selective resuscitation steps, parallel enrichments, or confirmatory testing on alternative media to improve recovery of stressed cells [30,36].

Additional blind spots arise when competing microorganisms outgrow the target organism during enrichment. Evaluations of EN ISO 11290-1 in mixed cultures, including newly described *Listeria* species, show that background microbiota can affect recovery of *L. monocytogenes*, reflecting conditions commonly encountered in real samples [117].

Detection outcomes are also influenced by media choice. Comparative studies show that some selective broths and agars inhibit growth of certain *Listeria* species or produce atypical colonies that complicate interpretation [118,119]. In such cases, negative results may reflect method performance rather than true absence.

Similar limitations are reported for other foodborne pathogens. For *S. enterica*, sublethal heat, oxidizing agents, and sanitizer exposure impair recovery, making detection highly dependent on enrichment and resuscitation conditions. This increases the risk of underestimation in complex foods such as chocolate, peanut butter, and poultry products [120,121,122,123,124]. Comparable effects are seen with *E. coli* O157:H7, where stressed cells remain viable but show reduced recovery on selective media. These findings show that culture-based blind spots reflect shared stress physiology rather than organism-specific traits [123,124,125,126,127].

### 6.2. Stress History and Detection Bias

Selective media can further suppress injured target cells while effectively inhibiting background flora [67,84]. Modeling studies show that injured cells are often more sensitive to selective components than uninjured cells, leading to underestimation when selective plating is used alone [128]. For this reason, some workflows include a nonselective resuscitation step before selective enrichment. Repair under nonselective conditions may be required before injured cells regain the ability to grow on selective media [84].

Stress history also introduces bias through enrichment dynamics. When recovery is slow or variable, only the fastest-recovering cells may reach detectable levels within fixed enrichment times, while slower-recovering survivors remain undetected [67]. This effect has been demonstrated in *Listeria* detection, where enrichment design directly influences which stressed cells are recovered [116,129]. Comparable enrichment bias has been reported for *Salmonella enterica* and *Campylobacter*. In *Salmonella*, sublethal stress and enrichment design influence recovery, with nonselective pre-enrichment improving detection of injured cells [120,121,123]. In *C. jejuni* and related thermotolerant species, cold, oxidative, and nutrient stresses common in poultry processing reduce culturability while maintaining viability. These conditions increase the likelihood of false-negative culture results [130,131,132,133].

Food-focused synthesis further shows that VBNC formation is linked to stress exposure under laboratory and controlled food-related conditions. Changes in temperature or nutrients can restore culturability without changing total viable cell numbers [19]. Because VBNC cells challenge culture-based detection, alternative viability methods are increasingly discussed. A 2024 review summarized viability-dye quantitative PCR approaches and highlighted both their strengths and their dependence on methodological choices [134].

Primary food studies demonstrate the practical value of these tools. Optimized PMA-qPCR protocols detected viable *Campylobacter* cells in chicken meat, including VBNC populations that were not detected by conventional culture in the same studies [135,136]. At the same time, culture-independent methods require careful interpretation. Conventional quantitative PCR amplifies DNA from dead and live cells, which can overestimate viable contamination unless viability dyes are applied appropriately [134,137].

### 6.3. Implications for Verification and Risk Assessment

Monitoring programs aim to verify sanitation and detect environmental harborage. Guidance documents emphasize environmental sampling and describe how pathogens such as *L. monocytogenes* can transfer from processing environments to RTE foods [138,139]. Stress-hardened bacteria complicate interpretation of negative results. Poor recovery of biofilm-associated cells, suppression of injured cells during selective enrichment, and VBNC states can all produce negative culture findings despite persistence of viable cells [67,109]. These effects describe detection limitations rather than confirmed transmission or illness risk in commercial settings.

These limitations support the growing emphasis on trend-based environmental monitoring rather than reliance on single results. Food and Drug Administration (FDA) guidance highlights environmental control as critical when products are exposed before packaging [138,139]. Regulatory programs have also evolved. FSIS directives describe expanded sampling strategies, and communications announced enhanced measures, including broader *Listeria* species testing in products and environments beginning in January 2025 [140,141]. This shift reflects recognition that genus-level data and environmental context improve verification.

Verification should be treated as a biological measurement, not only a procedural requirement. When stress-hardened states are plausible, monitoring benefits from methods and interpretation strategies that account for repair, delayed recovery, enrichment competition, and loss of culturability. Culture-based testing remains essential, but results should be interpreted in light of these biological processes, with complementary approaches applied when the risk context warrants them [67,117,134]. Table 2 supports a practical summary linking stress exposure to persistence outcomes and expected monitoring limitations, offering guidance for method selection and result interpretation.

## 7. Predicting Bacterial Behavior in a Stress-Driven Food Chain

Predictive microbiology is widely used to estimate microbial growth, survival, and inactivation for process design and verification [142]. In practice, many models rely on laboratory data generated under controlled and static conditions [142]. Real food chains, however, expose bacteria to changing environments and combinations of stresses during storage, processing, and sanitation. As a result, bacteria entering a control step often differ physiologically from the laboratory cultures used to generate predictive models [29]. When stress history is not represented, predictions may describe an idealized organism rather than the organism that actually encounters a control step [143,144]. This mismatch can lead to overestimation of control performance under real processing conditions.

A major limitation is that many predictive models are based on short-term experiments that do not account for cumulative exposure [142]. In real food systems, bacteria may experience refrigeration, acidification, drying, or oxidative stress before reaching a thermal or nonthermal intervention [67]. Reviews of predictive microbiology show that such sequential exposures can alter lag time, growth probability, and inactivation behavior, even when final processing conditions remain unchanged [145,146]. When these effects are not represented, models may underestimate survival following industrial treatments.

Another common assumption is that microbial populations behave uniformly under defined conditions. Increasing evidence shows that this assumption rarely holds, even within a single strain [147]. Reviews in predictive food microbiology describe cell-to-cell differences in growth, lag, and survival and explain how these differences generate variability that is not captured by average behavior. This variability helps explain why identical treatments can produce shoulders, tails, or unexpected survivors across replicate experiments [63,148]. For food safety decision making, this means that average model outputs may fail to capture the behavior of the most persistent cells [149].

Single-cell studies reinforce this view. Stress exposure often increases heterogeneity rather than reducing it [149]. Research on microbial phenotypic variability shows that genetically identical cells can differ markedly in stress tolerance and growth behavior because of stochastic differences in gene expression [148,150]. Reviews of single-cell measurement approaches further demonstrate that individual growth and lag times vary substantially under dynamic conditions. These findings show that a small fraction of cells can dominate survival outcomes, even when most of the population is inactivated [151,152].

Because heterogeneity affects outcomes, predictive microbiology has increasingly adopted frameworks that describe microbial behavior as a distribution rather than a single curve [148]. A review of variability in microbial inactivation explains how probability-based models and single-cell concepts better represent inactivation outcomes, particularly when risk depends on a small fraction of surviving cells. This perspective is especially relevant for food safety, where failure is often driven by rare survivors rather than average behavior [63,65].

Random variation further influences outcomes when survival probabilities are very low. Even in the absence of physiological differences, survival remains probabilistic [153]. Studies on the role of chance in microbial survival show that stochastic effects can shape survivor curves and tailing behavior independently of biological variability and experimental error [154]. These effects can leave a measurable probability of rare survivors even after extended treatments [65]. Ignoring this element of chance can create unwarranted confidence when interpreting validation data. Modeling approaches that integrate both biological variability and random chance therefore provide a more realistic description of survival behavior [155].

Dynamic processing further affects bacterial survival during treatment. Experimental studies show that nonlinear survival curves are not always explained by differences present before treatment. In some cases, acclimation during exposure contributes to tailing and upward curvature. This illustrates that resistance can develop during processing itself, complicating predictions based solely on initial conditions [61,144].

Recent studies of thermal and nonthermal processing confirm that resistance parameters estimated under static conditions may not apply when conditions vary over time [156]. Dynamic models that allow physiological state variables to evolve during treatment describe experimental data more accurately than models that assume fixed resistance [61,157]. For validation, this means that time and temperature profiles can be as important as nominal treatment intensity [158].

Physical properties of foods also influence predictive accuracy. A 2024 study combined bacterial inactivation modeling with heat-transfer analysis to predict survival of desiccation-stressed *Escherichia coli* O157:H7 in hamburgers. Predictions changed substantially when realistic temperature gradients and prior stress were incorporated into the same framework [159]. Similar coupled models applied to other foods show that local microenvironments can protect stressed cells and create survival niches not captured by bulk temperature or time measurements [160]. These findings demonstrate that both physiological state and food structure shape survival outcomes. Predictive accuracy therefore depends not only on biological parameters but also on representation of physical heterogeneity, including food microstructure, fat distribution, pore networks, and phase boundaries [38,161].

These findings directly affect how food safety decisions are made during process validation and risk assessment. A 2023 review describing the use of predictive microbiology in European Food Safety Authority (EFSA BIOHAZ) risk assessments emphasizes the need to explicitly address variability, uncertainty, and biological realism [143]. When stress history, dynamic exposure, and population heterogeneity are not considered, risk may be underestimated and control effectiveness overstated. In a stress-driven food chain, model credibility improves when stress history, dynamic exposure, and population heterogeneity are treated as expected features rather than exceptions [143].

## 8. Implications for Food Safety Management and Control Strategies

Stress hardening changes how validated controls perform in real food chains [29]. Many interventions are evaluated using healthy, fast-growing cells under stable laboratory conditions [162]. In practice, bacteria often reach a control step after earlier exposure to acid, cold, drying, oxidants, or mild heat. These prior exposures can activate repair systems and stress responses that alter survival during later control steps. As a result, treatment performance can vary even when process conditions are unchanged [163]. The same intervention may therefore perform differently depending on microbial history rather than treatment intensity alone [59,164].

Recent studies in food microbiology show that repeated mild stresses during production, processing, storage, and distribution are common rather than exceptional [29]. Exposure to cold, acid, osmotic stress, and oxidants that prime adaptive responses has been documented across many pathogens and food environments. These findings demonstrate that stress history is a major determinant of control outcomes in food processing systems [164,165].

### 8.1. Limits of Single-Hurdle Thinking and Static Validation

Process validation often assumes that microbial resistance is fixed at the start of treatment. Evidence from dynamic processing studies shows that this assumption can be unsafe [156,166]. During dynamic heat treatments, bacterial cells can adapt while heating is still underway. Resistance can therefore increase during the process rather than remain constant throughout treatment [60].

Additional studies using time-varying thermal and nonthermal profiles support this view. Resistance can change during treatment, not only before it begins. Survival curves cannot always be explained by initial variability alone. Adaptive responses triggered during exposure contribute to tailing and unexpected survival [61,157]. These effects describe inactivation behavior during processing, not post-process growth or illness risk.

These findings have clear implications for challenge testing. When validation relies only on unstressed cultures, survival of stress-exposed populations may be underestimated. Experimental work shows that sublethal stress increases resistance of *Salmonella enterica* to ultraviolet-C treatment on almonds and fresh-cut leafy greens. This increased resistance was linked to activation of stress-response systems [59].

Similar results have been reported for desiccation, starvation, and sublethal stress in *Salmonella*, *E. coli* O157:H7, and *L. monocytogenes*. In each case, prior stress altered inactivation behavior and survival outcomes. These studies demonstrate that validation results depend strongly on the physiological state of the test organism [25,59,167].

Validation should therefore include cells that reflect realistic food-chain histories. This includes stationary-phase and stress-conditioned populations. Treatment profiles should also reflect industrial time and temperature patterns rather than idealized step changes [60]. This approach aligns with current guidance that emphasizes biologically informed validation rather than reliance on static worst-case assumptions [143,145,148].

### 8.2. Sanitation and Environmental Control as Selection Pressure

Sanitation is intended to control contamination, but it can also act as repeated selection pressure when exposure is incomplete or biofilms protect cells [168]. Biofilms on food-contact materials create microenvironments where bacteria encounter oxidants and surfactants without full inactivation. Under these conditions, more tolerant subpopulations are more likely to survive [83,169].

In food-processing settings, the choice of sanitizer depends on which microbes need to be controlled, the type of surface being cleaned, whether biofilms are present, and how much organic residue is on the surface [36,78]. Other practical factors, such as how the sanitizer interacts with equipment materials, worker safety, and regulatory approval, also influence which products can be used [170].

Comparative studies of *S. enterica* and *L. monocytogenes* biofilms on stainless steel show that resistance to sanitizers depends on the type of sanitizer used, its concentration, and contact time. These findings help explain why sanitation strategies validated using free-living cells may underperform against surface-associated populations [83].

Broader experimental and review studies confirm that repeated sublethal exposure to disinfectants can select for increased tolerance and persistence traits [171,172]. Uneven sanitizer delivery, neutralization by organic matter, and protected niches often expose bacteria to nonlethal concentrations over time. Such conditions promote survival and, in some cases, reduced sensitivity to sanitizers under processing conditions [173,174,175,176].

Field-based studies reinforce this interpretation for *Listeria*. Investigations of isolates from meat-processing environments show that persistent strains are linked to biofilm formation and protected niches rather than repeated introduction from raw materials [77]. Similar patterns have been reported for *Salmonella* and *E. coli* in poultry and meat processing. In these environments, biofilms on conveyors, drains, and equipment act as continuous sources of contamination despite routine sanitation [177,178,179].

Control strategies should therefore prioritize prevention of harborage and early removal of immature biofilms. Conditions that repeatedly deliver low-dose exposure should be avoided. Poor coverage, short contact times, and high organic loads favor survival and reseeding of the environment [83].

### 8.3. Integrating Stress Biology into Monitoring, Decision Making, and Risk Assessment

Stress-hardened states can reduce culturability. For this reason, monitoring results should be interpreted as biological measurements rather than simple pass–fail outcomes. In this context, biological measurements reflect the physiological state of the detected cells, including stress history, injury, delayed recovery, or entry into VBNC states, rather than a direct measure of contamination absence or illness risk [30,86]. This is especially important when injury, delayed recovery, or VBNC formation is likely. Culture-based testing tends to detect the fastest-recovering cells rather than the most persistent ones [164]. This pattern reflects detection bias during verification, not confirmed absence or presence of illness risk [86].

Recent guidance on environmental monitoring emphasizes that negative results must be interpreted in context. Stress exposure history, sanitation performance, and niche ecology all matter. These programs are described as dynamic and risk-based, relying on trend analysis and regular reassessment rather than isolated test results [180,181,182].

Complementary viability-based methods can support decision making when culture methods are likely to underestimate viable cells [52]. A 2024 foresight review summarized viability-dye quantitative PCR approaches used in food analysis. The review highlighted both their strengths and their sensitivity to protocol design and matrix effects [134].

The goal is not to replace culture-based testing. Culture remains essential for isolate recovery and typing [112]. Instead, a tiered approach is appropriate. Viability-targeted methods can help interpret negative culture results in high-risk situations and guide targeted sanitation or intensified sampling when warranted [134].

Risk assessment frameworks increasingly recognize the value of biological realism beyond average growth and inactivation. Accounting for stress adaptation, persistence mechanisms, and detection bias improves hazard characterization and reduces overconfidence in control verification [63,143,183].

Overall, evidence reviewed here shows that controls perform best when they anticipate stress history and limit repeated sublethal exposure. Controls should be validated using cells and treatment profiles that reflect real processing conditions. Aligning interventions, monitoring, and decision making with the biological drivers of persistence improves control reliability across the food chain [59,60,83].

## 9. Implications for Risk Assessment and Food Safety Policy

Microbiological risk assessment is used to support decisions when evidence is incomplete and outcomes are uncertain [50]. International guidance describes a structured process that includes hazard identification, hazard characterization, exposure assessment, and risk characterization [184]. This guidance also states that the purpose, assumptions, and outputs of an assessment must be clearly defined and transparent [50,185]. These principles are widely applied. However, many assessments still rely on simplified descriptions of microbial behavior that do not fully reflect stress-conditioned populations encountered in food systems. This simplification mainly affects how survival and detection are represented, rather than serving as direct evidence of consumer risk.

Stress-hardened bacteria are relevant to risk assessment because they influence what is measured at each assessment stage [29]. When bacteria experience sublethal stress before a control step, survival is not determined by the treatment alone. Prior exposure can modify recovery, growth probability, and culturability [186]. This affects hazard characterization because resistance during processing and sanitation is not fixed. It also affects exposure assessment because stressed cells may be present but not detected when recovery is delayed or culturability is reduced [50,143]. In these situations, risk may be underestimated because models and verification data represent only the fastest-recovering fraction of the population.

Uncertainty and variability are already recognized as core elements of microbiological risk assessment [184]. Guidance from the Food and Agriculture Organization of the United Nations distinguishes variability from uncertainty and explains why both must be described to avoid false confidence in conclusions [187]. Guidance from EFSA treats uncertainty analysis as an essential part of scientific assessment and requires assessors to identify sources of uncertainty and explain their effect on conclusions [188]. Stress hardening fits directly within this framework. It increases variability through population heterogeneity and adds uncertainty because stress histories in real food chains are rarely known with precision [62]. It does so by influencing variability in survival and detectability, not by defining virulence or dose and response relationships.

Policy tools can either reveal or hide these biological effects. Microbiological criteria are used to judge food or lot acceptability based on the presence, absence, or level of microorganisms or their toxins. Codex guidance links these criteria to risk management goals and verification activities [187,189]. However, sampling and testing cannot prove absence. The International Commission on Microbiological Specifications for Foods has long emphasized that no sampling plan can guarantee that a pathogen is absent. End-product testing alone cannot ensure food safety, particularly when contamination is rare or unevenly distributed [190,191]. When stress-hardened states reduce recovery or alter culturability, these limitations become more pronounced, and negative results may reflect sampling probability or method performance rather than true biological absence.

This leads to an important policy implication. Binary compliance decisions are fragile when they rely on single time point tests that ignore recovery dynamics, stress exposure, and persistence in processing environments [192]. Risk-based approaches are stronger when test results are interpreted together with uncertainty statements. They are also stronger when sampling plans and criteria match the intended level of protection. Risk characterization should clearly state the biological assumptions built into models and methods [50,143,185]. This does not mean lowering standards. It means designing and interpreting policy in ways that reflect how stress-conditioned bacterial populations influence detection and risk estimates.

Recognizing stress-hardened bacteria as a routine feature of food systems supports a shift toward risk-informed regulation that better reflects survival and detection behavior during food production [44]. In this approach, uncertainty is described rather than ignored, and decisions are aligned with observed bacterial behavior across the farm-to-fork continuum. Such alignment reduces reliance on negative findings alone, improves realism in hazard and exposure characterization, and clarifies the evidence needed to support defensible policy decisions [50,188].

## 10. Future Directions and Research Priorities

Food systems expose bacteria to repeated nonlethal stresses during production, processing, storage, and sanitation. These stresses include changes in temperature, acidity, moisture, and exposure to disinfectants. Repeated exposure can alter bacterial physiology in ways that slow or prevent recovery during culture-based testing. It can also support persistence through biofilms and other hard-to-remove populations. Prior stress can further reduce control effectiveness by increasing tolerance during later treatments. Future research should therefore treat stress history as a core element of hazard characterization. Stress history directly affects detection, persistence, and control performance in real food environments [59,108,193].

### 10.1. Monitoring That Accounts for Stress History

Most routine microbiological testing relies on culturability, fixed enrichment times, and selective media. Extensive research shows that bacteria exposed to prior stress can enter sublethally injured or VBNC states. Recovery from these states is often slow. It also depends on stress type and strain characteristics. In food systems, injured cells may fail to grow on standard selective media. This can lead to underestimation of contamination or false-negative results [19].

A key research priority is the development of monitoring approaches that reflect variability in post-stress recovery. Single-cell and strain-level studies using *L. monocytogenes* show that heat stress and combined stresses can greatly extend lag time during enrichment. This reduces detection efficiency under standard protocols [109]. These effects are not unique to *Listeria*. Similar stress-related delays in recovery have been reported for *S. enterica*, *C. jejuni*, and pathogenic *E. coli* after thermal, oxidative, cold, and desiccation stress. This illustrates that stress-dependent recovery is a general feature of foodborne bacteria.

A second priority is the targeted use of viability-focused molecular methods when culture-based approaches are likely to miss viable cells. Reviews of viability dye-based quantitative PCR show that these methods can better reflect living populations when cells are injured or nonculturable. These reviews also stress the importance of protocol optimization and consideration of food matrix effects [134].

Evidence from poultry-associated hazards highlights the broader relevance of these tools. Optimized PMA-based quantitative PCR has detected viable, including nonculturable, *Campylobacter* in chicken meat. These findings show that conventional culture can underestimate contamination [135,194]. In a recent retail survey, culture and viability quantitative PCR were applied in parallel to chicken breast samples. This approach provided a more complete picture of viable *Campylobacter* at retail [195].

A third priority is to move beyond pass-or-fail interpretation of monitoring results. Monitoring should document not only whether a target organism is detected, but also conditions that affect recovery. These include prior sanitizer exposure, duration of cold storage, drying, and cleaning frequency. Such stress histories can delay or suppress recovery during enrichment. In these cases, negative culture results may reflect reduced recoverability rather than true absence [113].

The strong focus on *L. monocytogenes* in this area reflects regulatory priority and long-term surveillance in RTE environments. It does not reflect unique stress biology. Stress-history effects on recovery, detection, and persistence have also been demonstrated for *S. enterica*. In this organism, enrichment performance depends on prior stress and physiological state, and modified recovery protocols continue to be refined [196,197]. For *C. jejuni*, advances in viability-linked quantification, including digital PCR combined with viability dyes, further confirm that culturability alone may fail to capture viable cells under stress [198].

Key research needs therefore include validation of enrichment and plating protocols using cells with defined stress histories rather than only unstressed laboratory cultures [64,109]. They also include identification of situations in which viability PCR adds meaningful information beyond culture, particularly after oxidizing disinfectants, cold stress, and nutrient limitation [134,135]. Finally, decision frameworks are needed that integrate test results with stress exposure history and facility-level trends.

### 10.2. Longitudinal and Systems-Level Studies

Short-term experiments are essential for identifying mechanisms. However, persistence and recontamination in food systems develop over long periods. They result from repeated survival and reintroduction in production and processing environments. Evidence from food facilities shows that contamination can persist for months or years. This makes it necessary to follow bacterial populations over time and across connected locations to understand persistence pathways accurately [46,103].

Longitudinal whole-genome sequencing studies in meat processing environments have been especially informative for *L. monocytogenes*. These studies allow tracking of lineages over time and help distinguish introduction from persistence [199]. More recent work shows that genomic data must be interpreted alongside detailed environmental and operational information. Facility layout, sanitation practices, and stress exposure patterns strongly influence which strains persist [200,201].

Similar longitudinal approaches are increasingly applied to other pathogens. For *Salmonella*, whole-genome sequencing across poultry production and processing stages has identified key entry points and movement patterns within integrated systems [202]. For *Campylobacter*, large-scale longitudinal and cross-sectional sequencing at the abattoir level provides a practical model for system-wide studies in high-throughput environments [203]. These findings highlight the need to extend long-term, systems-level designs beyond *Listeria*-focused research.

Another priority is to link longitudinal sampling with measurements of physiological state. Stress hardening is not defined by strain identity alone. It also reflects whether cells are injured, slow growing, or embedded in biofilms. Studies of sublethal injury during food-relevant heat treatments show that recovery behavior can vary widely within a population and strongly influence survival and risk [87].

Systems-level study designs should treat the food chain as biologically connected. Conditions encountered upstream, such as temperature fluctuations and transport-related stress, can affect downstream recovery, tolerance to sanitation, and establishment of persistent niches in processing environments [46].

Research priorities include multi-month sampling campaigns that combine isolate sequencing with records of cleaning practices, sanitizer chemistry, temperature profiles, and downtime [109,201]. They also include tracking of background microbiota alongside pathogens to identify ecological conditions that precede persistence events [200,204]. Finally, predictive models are needed that allow resistance and recovery potential to change over time rather than assuming fixed behavior.

### 10.3. Integrating Microbial Ecology

In food environments, pathogens rarely exist alone. They coexist with resident microbiota that influence attachment, biofilm formation, and responses to sanitation. Studies limited to single-species biofilms risk underestimating the protection provided by mixed microbial communities.

Experimental work shows that multispecies biofilms formed by environmental microbiota can alter both biofilm development and sanitizer tolerance of *L. monocytogenes*. This includes reduced susceptibility to disinfectants such as benzalkonium chloride [205,206]. These findings are consistent with evidence that biofilm age and structure increase tolerance to cleaning and disinfection. They support evaluation of control strategies using established communities rather than newly formed single-species biofilms [47].

Community-level protection is not limited to *Listeria*. Biofilms containing *Salmonella* and *E. coli* on food-contact surfaces show increased resistance to control measures and higher cross-contamination risk [207]. In processing environments, intensive sanitation can reshape resident biofilms and influence later *Salmonella* colonization and stress tolerance. These findings show that sanitation acts as both a control measure and a selective pressure at the community level [208]. Additional work shows that repeated or sequential sanitizer treatments can reduce biofilm biomass formed by *E. coli* O157:H7 and *S. enterica*. This reinforces the need to test sanitation strategies against established biofilm structures rather than planktonic cells alone [209].

Microbial ecology also affects detection. During enrichment, background microbiota can outgrow or suppress stressed target organisms. Changes in community composition can therefore alter recovery outcomes. Microbial ecology should be treated as a core component of both contamination control and verification [210].

Research priorities include characterization of facility biofilm communities and identification of taxa that consistently co-occur with persistent pathogens [204]. They also include testing sanitizers and cleaning regimes against mixed-community biofilms derived from facility isolates under repeated low-level exposure [47,206]. Finally, longitudinal study designs should link community structure with pathogen recovery, persistence, and recontamination [200,204].

In brief, the central need across these areas is integration. Monitoring approaches should consider cell viability and recovery potential, rather than relying only on culturability measured at a single time point. Research must follow microbial behavior over time and through connected environments, not only in short laboratory experiments. Control strategies must be evaluated within the ecological context in which multiple pathogens persist. A research agenda grounded in stress history, longitudinal evidence, and microbial ecology is more likely to produce interventions that remain effective across the full farm-to-fork continuum.

## 11. Conclusions

Food systems expose bacteria to repeated nonlethal stresses during production, processing, storage, and sanitation. These stresses accumulate over time and shape how bacteria survive, recover, and respond to control measures. The resulting populations differ from the unstressed cells typically used in laboratory studies. As a result, viable cells may escape detection, contamination may persist in biofilms and protected niches, and control steps may perform less consistently than expected. Although this review draws on evidence from several foodborne pathogens, much of the mechanistic understanding comes from *L. monocytogenes*. This bacterium provides a well-studied model for examining how stress exposure affects persistence, detection, and control in food systems. Across studies, these outcomes appear repeatedly in connected ways, reflecting biological responses shaped by stress history across the farm-to-fork continuum. Persistence and recontamination are therefore better understood as biological processes that operate across interconnected food production and processing environments. They arise from repeated exposure to sublethal stress, variable recovery behavior, and interactions with the surrounding environment, rather than from single failures in hygiene, sanitation, or testing. Addressing these challenges requires food safety approaches that explicitly consider prior stress exposure, variability in recovery, and persistence within specific food-processing environments. Monitoring, validation, and risk assessment are more reliable when they reflect bacterial behavior under real processing and sanitation conditions. Accounting for stress history provides a more realistic basis for food safety evaluation and reduces overinterpretation of negative results.

## Figures and Tables

**Table 1:** How Listeria monocytogenes persists in food processing and RTE environments.

Persistence Feature	Description in *L. monocytogenes*	Relevance to Food Processing and RTE Foods	Key References
Biofilm formation	Cells attach to food-contact surfaces and form structured communities that resist cleaning and disinfectants	Biofilms allow repeated contamination of foods even after routine sanitation	[36,37]
Sublethal injury	Heat, acids, or sanitizers damage cells without killing them	Injured cells may escape detection but later recover and grow	[10,30]
Delayed growth	Stress exposure increases lag time before growth resumes	Fixed enrichment times may miss slow-recovering cells	[42,105]
VBNC state	Cells remain viable but do not form colonies on standard media	VBNC cells can evade routine culture-based testing	[17,31]
Slow-growing survivors	A small fraction of cells shows reduced growth after stress exposure	These cells can persist in processing environments and contribute to recontamination over time	[106]
Persistent strains	Some *L. monocytogenes* strains tolerate stress and sanitation better than others	The same strains can be recovered repeatedly from one facility over time	[47,107]

**Table 2:** Stress history, persistence states, and monitoring blind spots for *L. monocytogenes*.

Stress Commonly Encountered	Observed Persistence State	Effect on Monitoring	Key References
Cold storage/refrigeration	Delayed growth and extended lag phase	Slow recovery during enrichment may cause false-negative culture results	[10,36]
Acid, salt, or osmotic stress	Sublethal injury and cross-protection	Injured cells may not grow on selective media without resuscitation	[29,30]
Repeated sanitation and surface contact	Biofilm formation and surface-associated persistence	Biofilm cells are less susceptible to sanitizers and harder to detect	[37,47]
Oxidizing disinfectants (e.g., chlorine)	VBNC state observed under laboratory models	Viable cells may escape culture-based detection	[31]
Selective enrichment with competing flora	Suppressed recovery during enrichment	Target cells may be outgrown and remain undetected	[110,111]

## Data Availability

No new data were created or analyzed in this study.
